# Soft tissue displacement for detection of left ventricle apical dyskinesis with transthoracic echocardiography

**DOI:** 10.1007/s10554-023-02856-4

**Published:** 2023-05-15

**Authors:** Brandon H. Schwartz, Balaji K. Tamarappoo, Hezzy Shmueli, Robert J. Siegel

**Affiliations:** 1 Department of Medicine, Cedars Sinai Medical Center, Los Angeles, CA USA; 2 Cardiovascular Institute, Indiana University School of Medicine, Indianapolis, IN USA; 3 Department of Cardiology, Soroka University Medical Center, Faculty of Health Sciences, Ben Gurion University of the Negev, Beer Sheva, Israel; 4 Cedars Sinai Medical Center, Smidt Heart Institute, 127 S. San Vicente Ave. A-3600, Los Angeles, CA 90048 USA

**Keywords:** Transthoracic echocardiogram, Wall motion abnormalities, Dyskinesis, Cardiac MRI, Soft tissue displacement

## Abstract

We tested the hypothesis that the use of outward displacement of the soft tissue between the apex and the chest wall as seen in TTE, is a sign of apical displacement and would allow for more accurate diagnosis of apical dyskinesis. This is a retrospective study of 123 patients who underwent TTE and cardiac magnetic resonance imaging (MRI) within a time frame of 6 months between 2008 and 2019. 110 subjects were deemed to have good quality studies and included in the final analysis. An observer blinded to the study objectives evaluated the echocardiograms and recorded the presence or absence of apical dyskinesis. Two independent observers evaluated the echocardiograms based on the presence or absence of outward displacement of the overlying tissue at the LV apex. Cardiac MRI was used to validate the presence of apical dyskinesis. The proportion of studies which were identified as having apical dyskinesis with conventional criteria defined as outward movement of the left ventricular apex during systole were compared to those deemed to have dyskinesis based on tissue displacement. By cardiac MRI, 90 patients had apical dyskinesis. Using conventional criteria on TTE interpretation, 21 were diagnosed with apical dyskinesis (23.3%). However, when soft tissue displacement was used as the diagnostic marker of dyskinesis, 78 patients (86.7%) were diagnosed with dyskinesis, p < 0.01. Detection of displacement of soft tissue overlying the LV apex facilitates better recognition of LV apical dyskinesis.

**Supplementary Information:**

The online version contains supplementary material available at 10.1007/s10554-023-02856-4.

## Introduction

The evaluation of wall motion of left ventricular (LV) segments is an integral component of cardiac imaging. During systole, the myocardium contracts and thickens inwards toward the ventricle cavity. In normal functioning myocardium, the LV motion is synchronous. American Society of Echocardiography (ASE) guidelines recommend visually assessing the LV regional function on transthoracic echocardiography (TTE) using a 17-segment model and grading abnormal wall motion qualitatively using a range of severity from normal to hypokinetic (reduced thickening), akinetic (absent thickening), dyskinetic (systolic thinning or stretching), and aneurysm (apical outpouching) [1]. Dyskinesis is used to describe ventricular muscle segments that bulge outside the LV cavity during systole, as opposed to contracting inwards with the rest of the segments.

The left ventricular apex is usually affected in disease states such as ischemic heart disease with LV apical infarction, hypertrophic cardiomyopathy, and Takotsubo cardiomyopathy. On physical exam, LV apical dyskinesis may be detected as systolic apical displacement during palpation. Although wall thickening of most LV segments can be assessed by transthoracic echocardiography, complete visualization of the LV apex is often difficult and confounded by foreshortening of the LV apex. This is due to nearfield artifact from the proximity of the transducer to the LV apex. These factors may result in underestimation of apical akinesis and dyskinesis by two-dimensional TTE. The outward movement of soft tissue overlying the LV apex seen in patients with apical dyskinesis in the apical 2- and 4-chamber views is associated with infarctions due to left anterior descending (LAD) artery occlusion due to involvement of the blood supply affecting the apex and its proximity to surrounding structures, including the pericardial fat pad, which can transmit dyskinetic segments [2].

Dyskinesis is associated with an infarcted myocardial segment and is more commonly seen at the LV apex. The prevalence and characteristics of apical dyskinesis following myocardial ischemia and necrosis, however, is not well described. Our objective was to test the hypothesis that the use of soft tissue displacement can be identified by TTE (analogous to the findings in physical exam) in the apical views, improving the accuracy of detecting apical dyskinesis in patients suspected to have apical wall motion abnormalities.

## Methods

We performed a computerized search on all cardiac MRI and TTE studies performed at Cedars-Sinai Medical Center (CSMC) in Los Angeles, CA, USA, which contained the diagnosis of “apical dyskinesis” in the report. All clinical and imaging data were collected from the electronic medical records. The study met ethical requirements set forth by the Institutional Review Board and approved at Cedars Sinai Medical Center.

**Fig. 1:**
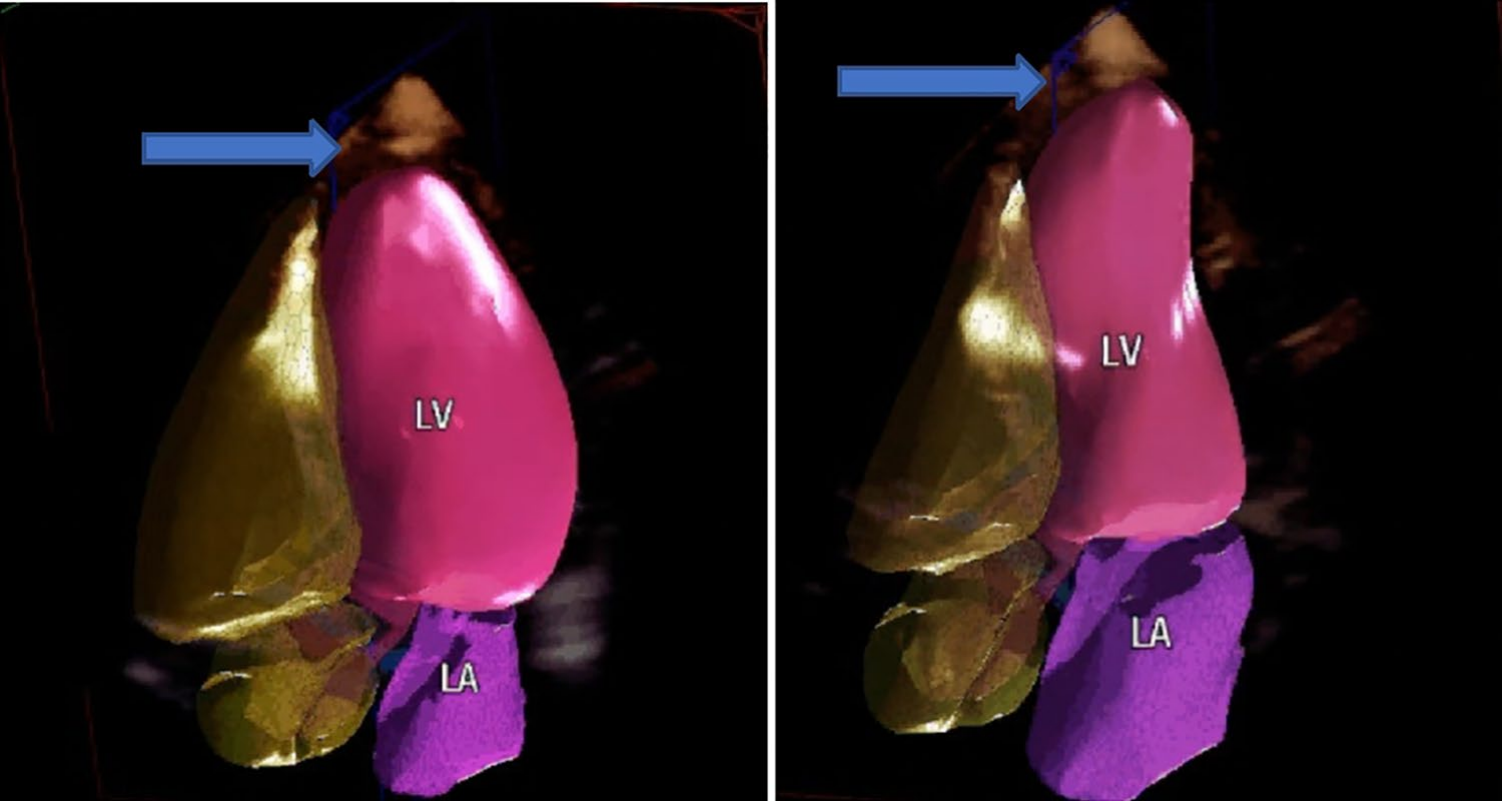
Three-dimensional transthoracic echocardiographic images of dyskinesis seen during diastole (left) and systole (right). Soft tissue overlying the left ventricle (LV) demarcated by arrows with displacement noted at the apex

Patients who underwent TTE and cardiac MRI within 6 months at CSMC from 2008 to 2019 were included in the study. The time delay between the two studies was < 6 months with average delay being 21 days (range 1–154 days). 103 patients with apical dyskinesis on MRI were initially reviewed; however, 90 were selected as having decent visualization of the LV apex. An additional 20 patients with normal LV ejection fraction, no history of ischemic or nonischemic cardiomyopathy and who underwent cardiac MRI for evaluation of the etiology of premature ventricular beats were selected as controls (Fig. 1).

### TTE

TTE was performed for clinical purposes per standard protocol using standard ultrasound scanners (Philips iE33; Philips Medical Systems; GE Vivid E9, GE Healthcare). Wall motion was assessed with a standard 17-segment model using apical 4-, 3-, and 2- chamber view. Scoring was based on visual assessment of motion of LV segments and apical cap. As the study exams took place before longitudinal strain and contrast echo were used as standard methods to better evaluate the LV apex, it could not be included as part of our evaluation. Apical wall motion was graded by standard echocardiographic analysis on echo reports. The same studies were reviewed by the authors for presence of outward motion of the soft tissue overlying the LV apex (Videos 1, 2). Additional three-dimensional (3D) TTE images were obtained in one example to better illustrate the presence of soft tissue displacement overlying the apex in diastole versus systole (Fig. 1).

### Cardiac MRI

CMRI was performed as per standard protocol for a variety of cardiac reasons using a 1.5-Tesla scanner (Avanto, Siemens Healthcare, Erlangen, Germany), with electrocardiogram gating and phased-array surface coil (CP Body Array Flex; Siemens Healthcare), and subjects in the supine position. Steady state free precession images were obtained with axial, vertical long axis and three chamber views. Short axis stack of SSFP cine images was obtained with post-processing LV apical dyskinesis was assessed by a level 3 certified CMRI reader. LV ejection fraction was measured using Circle CVI42 software.

### Statistical analysis

Descriptive statistics were summarized as mean or number with percentage. Ejection fraction measurements were further evaluated using mean, median, and interquartile range. Analysis was obtained by Cohen’s kappa statistic using a table comparing initial echocardiographic evidence of dyskinesis to reclassification of dyskinesis on echo read. This statistic is used to define inter-rater reliability for qualitative items. Negative numbers indicate no agreement and the closer the number to 1, the more congruent the observers. Additional evaluation with Fisher’s exact test using a contingency table with p-values were subsequently obtained with significance defined as p < 0.05.

## Results

### Patient characteristics

**Fig. 2:**
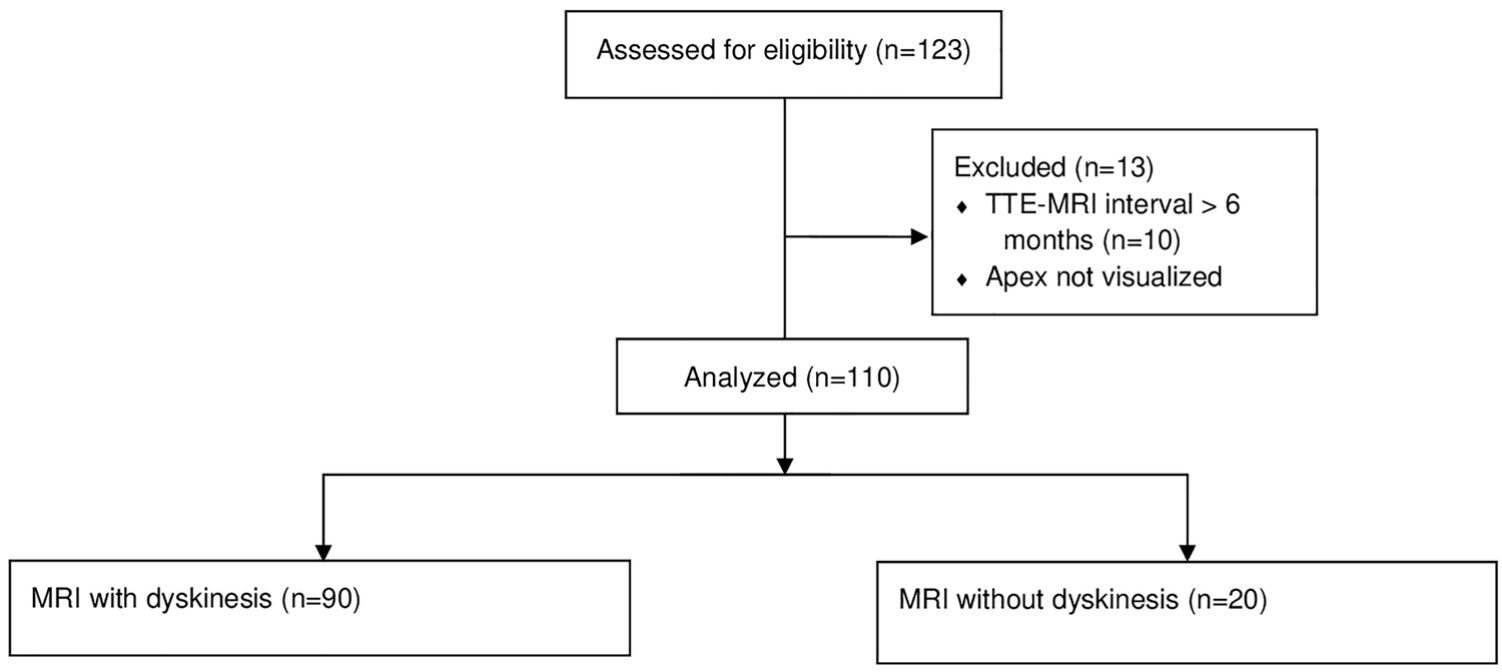
Flow chart demonstrating patient selection. 123 patients at Cedars-Sinai Medical Center were assessed for inclusion in the study. 13 were excluded for not meeting criteria including well-visualized apex and TTE-MRI interval within 6 months of each other. 90 of the 110 patients had MRI evidence of dyskinesis

Out of the initial 123 participants that were selected as having both TTE and MRI, 110 patients selected for analysis with 90 having MRI-proven dyskinesis and 20 control patients without dyskinesis on MRI (Fig. 2). 84 (76.4%) were men with an average age of 58.3 ± 12.6 years old and a BMI of 26.5 ± 5.2 kg/m^2^, with an LV ejection fraction of 40.7 ± 16.5% (Table 1). The major indication for echocardiography was either heart failure or coronary artery disease and the major indication for MRI was either myocardial viability or cardiomyopathy.

**Table 1:** Clinical characteristics of patients evaluated by both TTE and MRI

	MRI proven dyskinesis	MRI without dyskinesis
Age (average), in years	59.6 ± 11.9	53.0 ± 14.7
Sex		
Male	72 (75.8%)	12 (60.0%)
Female	23 (24.2%)	8 (40.0%)
BMI (average), in kg/m2	26.8 ± 5.4	25.4 ± 4.1
Echocardiogram indication		
Heart failure	38	
Coronary artery disease	22	20 patients selected with premature ventricular contractions undergoing evaluation with MRI
Stroke/embolization	6	
Chest pain	6	
Pericardial effusion	5	
LV thrombus	4	
Valvular heart disease	4	
Shortness of breath	3	
Pulmonary hypertension	2	
Ejection fraction		
Mean EF	36.3%	60.7%
Median EF	36%	61%
Interquartile range	Q3 (45) − Q1 (25) = 20%	Q3 (64) − Q1 (59) = 5%
Patients with ICD/pacemaker	2	0

### LV dyskinesis assessment

**Fig. 3:**
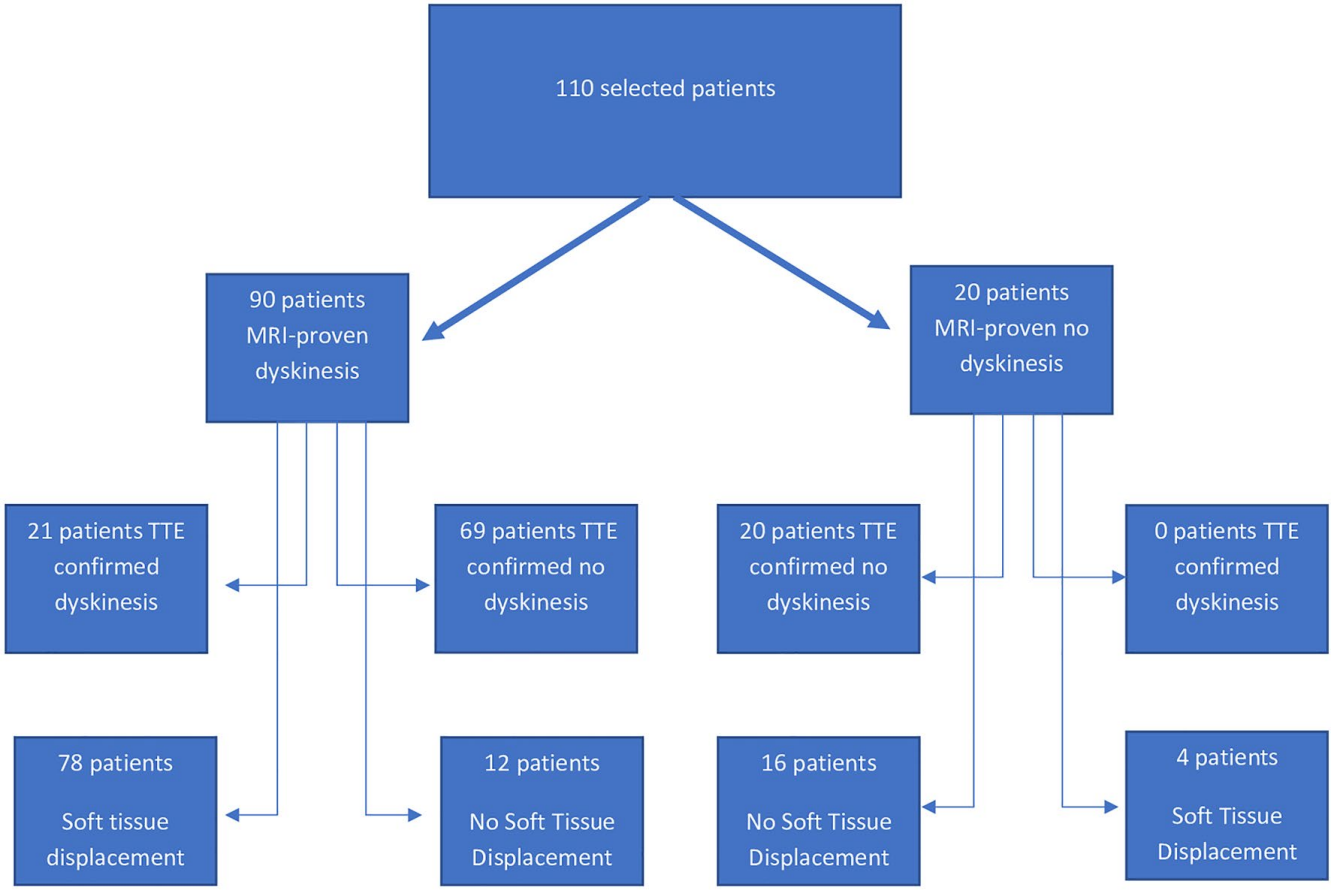
Flow chart showing interpretation of selected patients using both standard echocardiographic analysis and soft tissue displacement analysis

**Fig. 4:**
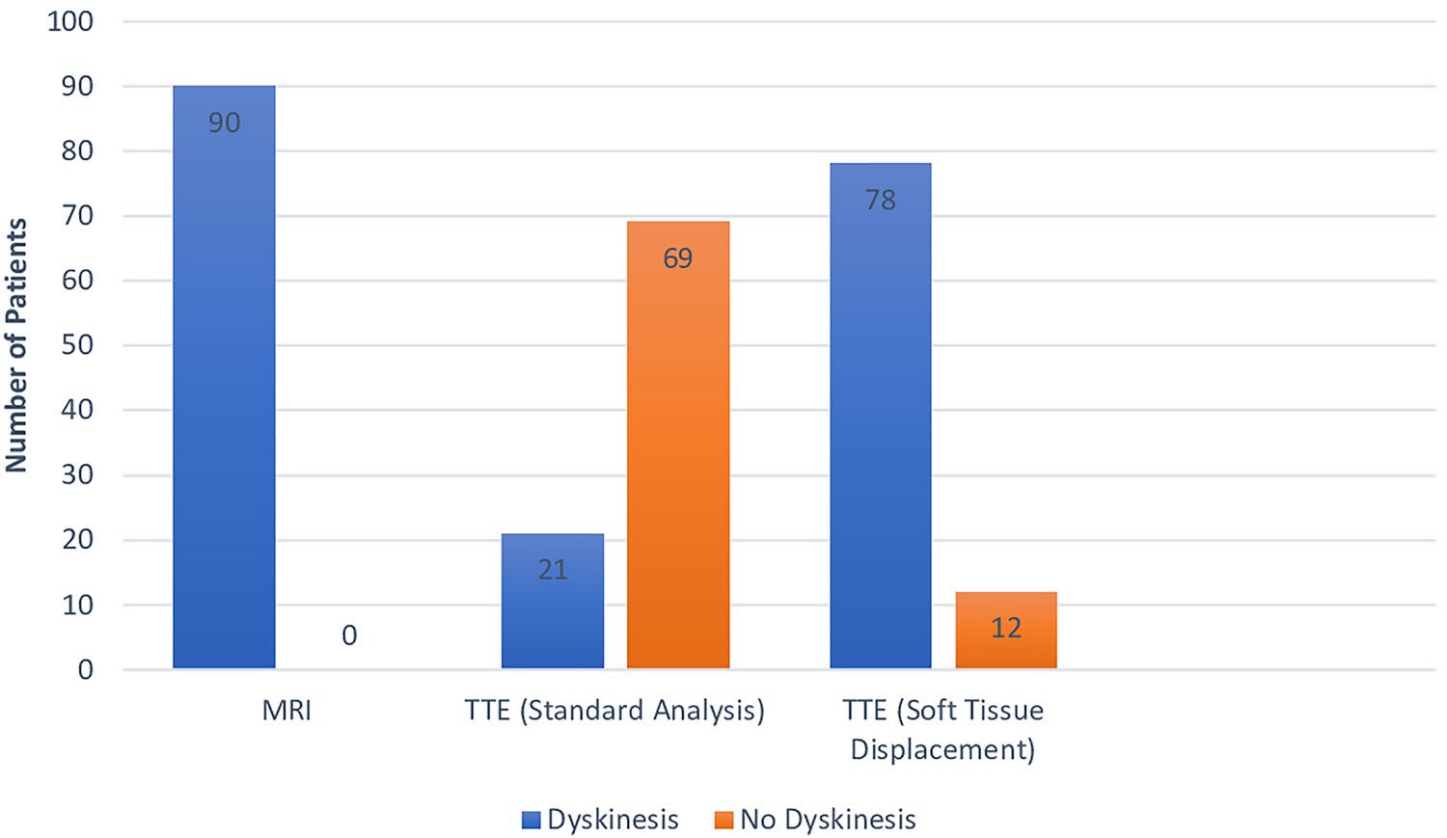
Bar graph evaluating dyskinesis results based on standard criteria and with new soft tissue displacement criteria. Y-axis demonstrates number of patients meeting criteria. 69/90 (76.67%) without dyskinesis. 21/90 (23.33%) with dyskinesis on initial interpretation. 12/90 (13.33%) without dyskinesis. 78/90 (86.67%) with dyskinesis with reclassification criteria. K = -0.32, p-value < 0.01

Of the 90 patients selected with MRI evidence of apical dyskinesis, 21 (23.3%) had dyskinesis on the initial formal echo read. However, after reclassification with interpretation of soft tissue displacement, 78 (86.7%) were found to have dyskinesis (Fig. 3). This difference was associated with a Cohen’s kappa value of -0.32 indicating no agreement between the initial echo read and reclassification. Additionally, using Fisher exact test, this difference was associated with a p-value of < 0.01 by chi-square analysis (Fig. 4).

In the control arm with MRI indicating no LV dyskinesis, 4 of the 20 subjects had displacement of the LV apex on TTE.

## Discussion

In this retrospective study, we report that patients with apical dyskinesis were more readily identified by using the displacement of the soft tissue overlying the LV apex. This is the first study evaluating this novel diagnostic measure for detecting LV apical dyskinesis.

Apical dyskinesis is commonly observed in ischemic cardiomyopathy and is conventionally diagnosed by visual assessment of LV apical wall motion. In addition to ischemic heart disease, apical wall motion abnormalities can occur in Takotsubo cardiomyopathy, apical hypertrophy, RV apical pacing, vasospastic angina, and nonischemic dilated cardiomyopathy [3–5]. Wall motion assessment of the LV apex can be fraught with inaccuracies due to incomplete visualization of the true apex and near field artifacts. While incorporating contrast in wall motion analysis has been reported to improve interobserver agreement of segment visualization in global LV function, abnormalities of apical wall thickening remain underestimated, and their clinical significance may be underappreciated [6]. Cardiac MRI is used as the gold standard modality for assessment of LV wall thickening and volumetric assessment of LV and right ventricular function due to its superior endocardial definition and comparable spatial and temporal resolution when compared to TTE. MRI also allows optimal visualization of the LV apex [7]. We found that 87% of patients were correctly reclassified as having LV dyskinesis on TTE using the criteria of LV apical soft tissue displacement when compared to the initial TTE interpretation. Translational apical wall motion has been used as a measure of dyssynchrony in patients who are being evaluated for cardiac resynchronization therapy [8].

In ischemic cardiomyopathy, LV apical wall motion abnormalities may have prognostic importance as regional LV dyskinesis is associated with adverse cardiovascular outcomes in the first 30 days after a myocardial infarction (MI) [9]. Additionally LV thrombi are more likely to occur in dyskinetic or aneurysmal segments and seem to disappear less often than in akinetic areas [10].

In the control arm with MRI indicating no LV dyskinesis, 4 of the 20 subjects had displacement of the LV apex on TTE. Of note, 3 of those had moderate aortic regurgitation and ascending aortic root dilation, which may have contributed to finding of soft tissue displacement. The remaining patient had evidence of apical-variant hypertrophic cardiomyopathy.

## Limitations

This is a single-center retrospective study of patients who underwent CMRI and TTE for clinical indications, which were different for each patient. Furthermore, the TTE and CMRI were not performed specifically for identifying apical dyskinesis which could affect windows and views. As this is an observational study the authors do not know the exact mechanism behind why the apical dyskinesis is present and further studies using our findings should be undertaken to better understand the significance.

As noted above, there was evidence of soft tissue displacement in the echocardiograms of 4 patients without dyskinesis on MRI. It is not clear to the authors why this may be the case but it is important to note that while this novel finding may increase the sensitivity of determining dyskinesis, it may negatively affect specificity.

## Conclusion

In this study we report a novel transthoracic echocardiographic finding (displacement of LV apical soft tissue) that facilitates the detection of left ventricular apical dyskinesis.

## Supplementary Information

Below is the link to the electronic supplementary material.

Video 1: Video demonstrating soft tissue displacement of a 2-chamber view transthoracic echocardiogram (TTE). Supplementary file1 (MP4 632 kb)

Video 2: Video demonstrating soft tissue displacement of a 4-chamber view transthoracic echocardiogram (TTE). Supplementary file2 (MP4 746 kb)

## Abbreviations

LV: Left ventricle
TTE: Transthoracic echocardiography
MRI: Magnetic resonance imaging
BMI: Body mass index
EF: Ejection fraction
ASE: American Society of Echocardiography
LAD: Left anterior descending
MI: Myocardial infarction
CSMC: Cedars-Sinai Medical Center
